# What topics should we teach the parents of admitted neonates in the newborn care unit in the resource-limited setting - a Delphi study

**DOI:** 10.1186/s40748-019-0106-8

**Published:** 2019-07-11

**Authors:** Jean Aime Musabyemungu, Alice Willson, Sean Batenhorst, James Webbe, Peter Thomas Cartledge

**Affiliations:** 1 University of Rwanda, Kigali, Rwanda; 2 University Teaching Hospital of Kigali (CHUK), Kigali, Rwanda; 3 Rwanda Human Resources for Health (HRH) Program, Yale University (USA), Kigali, Rwanda; 4 Royal College of Paediatrics and Child Health, UNICEF neonatal programme, Kigali, Rwanda; 5 University of Wyoming, Laramie, Wyoming USA; 6 Imperial College London, London, UK

**Keywords:** Education, Caregiver, Infant, newborn, Developing countries

## Abstract

**Background:**

In resource-limited settings, such as Rwanda, health care profession (HCP) to neonate ratios are low, and therefore caregivers play a significant role in providing care for their admitted neonates. To provide such Family Integrated Care, caregivers need knowledge, skills, and confidence. The objective of this study was to identify consensus from key stakeholders regarding the priority topics for a “parental neonatal curriculum.”

**Methods:**

A three-round Delphi-study was conducted. During Round-1, face-to-face interviews were undertaken and responses coded and categorized into themes. In Round-2, participants were presented with Round-1 feedback and asked to provide additional topics in respective themes. In Round-3, respondents were asked to rank the importance of these items using a 9-point Likert scale.

**Results:**

Ten, 36 and 40 stakeholders participated in Rounds-1, − 2 and − 3 respectively, including parents, midwives, nurses and physicians. Twenty and 37 education topics were identified in Rounds-1 and -2 respectively. In Round-3 47 of the 57 presented outcomes met pre-defined criteria for inclusion in the “parental neonatal curriculum.”

**Conclusion:**

We describe a “parental neonatal curriculum,” formed using robust consensus methods, describing the core topics required to educate parents of neonates admitted to a newborn care unit. The curriculum has been developed in Rwanda and is relevant to other resource-limited settings.

**Electronic supplementary material:**

The online version of this article (10.1186/s40748-019-0106-8) contains supplementary material, which is available to authorized users.

## Introduction

The majority of neonatal deaths occur in two regions of the world: 39% in sub-Saharan Africa and 38% in Southern Asia with 99% of neonatal mortality being found in the resource-limited setting [1]. Worldwide neonatal mortality has declined slower than other rates of under-5 mortality [2, 3]. Rwanda is committed to meeting Sustainable Development Goal (SDG) 3.2.2: ending preventable deaths of newborns and children under 5 years of age by 2030 [4]. In order to achieve this care facilities in low-income countries (LICs) should deliver proven, effective interventions to substantially reduce newborn mortality [5]. Monitoring and evaluation of interventions and care programs are vital to identify effective interventions [6]. The Rwandan Ministry of Health includes a Neonatal Working Group to implement such change nationally and data monitoring is undertaken using the Integrated Health Management Information System (HMIS) [7].

In resource-limited settings, such as Rwanda, nurse to neonate ratios are low. Family Integrated Care (FICare) is frequently employed as a necessity, integrating parents as primary caregivers of their sick newborns [8, 9]. Care provided by parents to their admitted neonate is dependent upon knowledge, practice, and confidence of the parents regarding neonatal care and may go on to determine the neonate’s health status and length of neonatal admission. It is possible that in the resource-limited setting that there is a contribution to morbidity and mortality due to an inadequate parental understanding of neonatal illness and the care that these newborns require [10]. Therefore, equipping parents with proper knowledge of essential neonatal care could contribute to improving the outcomes of these neonates [10–13].

The admission of a sick neonate is a stressful period for parents [14, 15] and so education of parents is paramount to not only improve the quality of care they provide but also to reduce the stress of caregiving and enhance confidence at the point of discharge [16, 17]. As the number of HCPs is low, priority should be given to the key topics to ensure maximum benefit for the neonate without overburdening HCPs with educational roles.

Parental education could include topics such as immediate and exclusive breastfeeding, hand washing, management of hypothermia, hygienic cord cleaning, recognition of danger signs for neonatal illness and kangaroo mother care (KMC) for low birthweight (LBW) neonates [2, 18]. It has been shown that parental education is associated with improved neonatal care practices in well, non-admitted neonates [19, 20]. Currently there is no consensus about what topics parents of sick, admitted, neonates should be taught in this setting.

## Methodology

### Aims

To identify consensus from key stakeholders regarding the priority topics for a “parental neonatal curriculum.”

### Scope

This “parental neonatal curriculum” describes the core topics to educate parents of neonates admitted to a neonatal unit in Rwanda, and would also be applicable in other resource-limited settings.

### Study design

This was a three-round Delphi study. Delphi methods use sequential “rounds,” with controlled feedback between rounds to build consensus from a group of experts [21]. The Delphi method is useful in situations where individual opinions and judgments need to be considered and combined to answer an incomplete state of knowledge. The process was “fully anonymized,” that is participants did not know the identities of the other individuals in the group, nor did they know the specific answers that any other individual had given.

### Participants

We recruited two groups:

- *Group 1 - Parents:* Parents of admitted neonates were eligible for inclusion. Parents of neonates with a poor prognosis where participation could be distressing for the participant were excluded along with parents who were themselves under 18 years-of-age. Parents were recruited at two newborn care units of the University Teaching Hospital Kigali (CHUK), and Muhima District Hospital (MDH). Due to the transient nature of parents at the two sites, the parent participants were different in each Round of the Delphi study. Convenience sampling was employed at the clinical sites.

Both units are found in Kigali, the capital city of Rwanda. CHUK is a tertiary level hospital with the newborn unit has approximately 560 admissions and caters for 20–30 infants every day, with three Kangaroo Mother Care (KMC) spaces. The obstetric department is a referral unit and the principal site for approximately 2000 high-risk deliveries per year [22]. MDH is a district hospital, located in Kigali city, and serves approximately 1 million people. The hospital has only two major departments: obstetrics & gynecology and pediatrics with neonatology and is responsible for approximately 15,000 deliveries per year. The MDH neonatal unit includes 25 cot spaces and eight KMC spaces. Both neonatal units would be considered a level II by USA standards [23] and level I by UK standards [24], providing simple therapies such as CPAP and intravenous fluids, without mechanical ventilation or total parental nutrition. There are no admission weight cut-offs, and standard practice requires a weight of 1.8 kg before discharge [22].

- *Group 2 - Expert stakeholders:* We defined an “expert” as professionals who had experience in clinical care for neonates and their families in a resource-limited setting, such as Rwanda. These experts were drawn from the following: (i) Nurses and midwives at the two clinical sites; (ii) Rwandan clinicians and residents working in Rwanda in pediatric and neonatal care who were identified via the pediatric academic faculty at the University of Rwanda; (iii) Members of the Rwandan Ministry of Health (MoH) Neonatal Working Group (NWG) including pediatricians, nurses and midwives, identified through the chair of the NWG; (iv) General Practitioners (clinicians working in district hospitals) identified through the class-representatives at the University of Rwanda; (v) Non-Rwandan, international pediatricians and neonatologists with experience of working in Rwanda through the Human Resources for Health (HRH) program [25] identified from the Ministry of Health (MoH) database of HRH faculty. We communicated with the expert stakeholders by e-mail or via visiting the two clinical sites.

### Sample size

In Round-1 we aimed to undertake face-to-face interviews with 10 participants. For Rounds-2 and -3, we aimed to gain responses from a minimum of 15 respondents, in each round, which is considered the required number for achieving consensus in Delphi studies [26]. Group-2 response rate was predicted to be 10%, therefore, invites were sent to 80 potentially eligible participants. Non-participation in Round-2 did not exclude participation in Round-3. New participants in Group 2 were not added between Round-2 and -3. The exception to this were nurses and midwives at the clinical sites who were recruited opportunistically at the clinical sites and completed paper rather than online questionnaires.

### Questionnaire development

The questionnaires were designed specifically for the purposes of this study. The questionnaires and feedback were translated for parents into Kinyarwanda, the single unifying language of Rwanda, by the Principal Investigator (JAM). Expert stakeholders (Group-2) completed the questionnaire in English. All questionnaires were piloted for understanding before use. Paper questionnaires (face-to-face) were administered at the clinical sites for parents, nurses and midwives (CHUK and MDH) and therefore this group of stakeholders did not require internet access. Electronic questionnaires (Google Forms®) were sent by email to expert stakeholders (Group-2) found outside of the clinical sites.

### Consensus process

Participants took part in three rounds of surveys.

#### Round-1 (oral open-questions)

Face-to-face interviews were employed to build an initial draft list of the “parental neonatal curriculum” topics. Two open questions were posed (see Additional file 1) for participants to describe the topics. As topics were identified using interviews there was no word limit on responses. The questions were asked verbally with responses collected by the PI using field notes. No voice recordings of the interviews were undertaken. Parents responses were then translated by the PI. HCPs responded in English. The responses were then coded, and summarised in Microsoft Excel by the PI (JAM) and supervising consultant (PC). Consensus for inclusion in Round-2, was pre-defined as any topic suggested by any one participant. The initial list of topics was categorized into five domains.

#### Round-2 (free-text open-questions)

Feedback from Round-1 was given to participants, with all the topics generated in Round-1 being presented to participants in the questionnaire. The items were presented within each individual domain and participants were then asked to add any additional topics that they felt were missing, within that domain, and should be added to the curriculum (see Additional file 2: for questionnaire). Parental responses were translated to English by the PI. HCPs responded in English. Responses were then coded and analyzed in Microsoft Excel. Duplicate items from Round-1 were removed. Consensus for new items to be included in Round-3 was pre-defined as any single topic that was given by any one participant.

#### Round-3 (closed-questions)

Feedback was given to participants with the items from Round-1 and -2 being combined in a single list, and by presenting each topic with feedback in the form of a percentage of participants who had suggested it. These items were presented to parents and expert stakeholders who were asked to grade the importance of the topics using a 1–9 point Likert scale as described by the GRADE development group [27, 28]. The data-collector (JAM) presented the list to parents and was available to clarify any items that parents did not understand. Consensus for inclusion in the final “parental neonatal curriculum” was pre-defined as items with greater than 70% of participants scoring 7–9 (important) AND less than 15% of participants scoring 1–3 (not important) [28].

### Correlation of importance of topics between stakeholder groups

To assess for overall correlation in opinion between the three stakeholder groups comparison of the mean scores of each topic was undertaken using linear regression and Pearson’s correlation (R). The importance of each individual topic was categorized into three levels of importance, namely 7–9 (important), 4–6 (intermediate) and 1–3 (not important) and then each individual item was compared between subject groups (clinicians, nurses, caregivers) using Chi-squared. Each item was color coded for importance with green representing high importance and red reflecting low importance. This allows for a visual comparison between the stakeholder groups.

## Results

No deviation in the original study protocol was required. Reporting of this study is per the COS-STAR checklist for Delphi studies [29].

### Participants

**Fig. 1:**
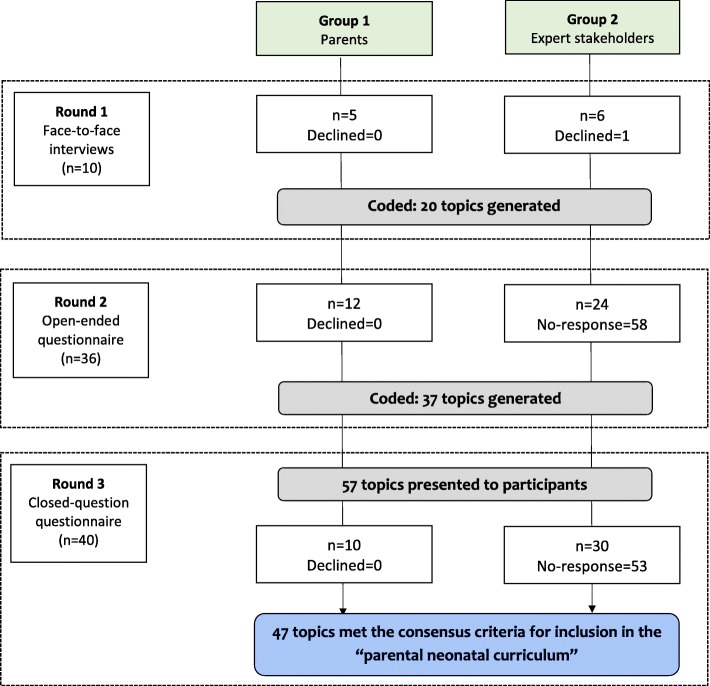
Study flow-chart

Ten, 36, and 40 participants took part in Rounds-1, − 2 and − 3 respectively (Table 1 and Fig. 1). The overall response rate was 91, 38 and 43% respectively which far exceeding our predicted response rate. The overall response rate was 100% amongst parents and 35% (59/171) in the experts. Expert stakeholders were from four countries; Rwanda, USA, United Kingdom, and Tanzania. There was no mechanism to measure any challenges with access in stakeholders receiving the questionnaire electronically. Parents were a key group within our stakeholders, representing 50, 33 and 25% of participants in Rounds-1, − 2 and − 3 respectively (Table 1).

**Table 1:** Baseline characteristics of participants of Rounds 1–3

			Round-1 (*n* = 10)	Round-2 (*n* = 36)	Round-3 (*n* = 40)
All	Response rate	All	10/11 (90.9%)	36/94 (38.2%)	40/93 (43.0%)
		HCPs	5/6 (83.3%)	24/82 (29.3%)	30/83 (36.1%)
		Parents	5/5 (100%)	12/12 (100%)	10/10 (100%)
	Questionnaire administration	Electronic	NA	22 (61.1%)	26 (65.0%)
		Paper		14 (38.9%)	14 (35.0%)
	Role	Parent	5 (50.0%)	12 (33.0%)	10 (25%)
		Pediatricians	3 (30%)	13 (36.1%)	20 (50%)
		General Practitioner	0 (0.0%)	6 (17.0%)	4 (10.0%)
		Nurses and midwives	2 (10.0%)	5 (13.9%)	6 (53.0%)
	Age	20–29	5 (50.0%)	11 (30.6%)	12 (30.0%)
		30–39	4 (40.0%)	21 (58.3%)	23 (57.5%)
		> 40	1 (10.0%)	4 (11.1%)	1 (2.5%)
	Gender	Male	1 (10.0%)	17 (47.2%)	16 (40.0%)
		Female	9 (90.0%)	19 (52.8%)	24 (60.0%)
HCPs	HCPs Main place of work	Rwanda	5 (100%)	21 (88.0%)	26 (87.0%)
		USA	0 (0%)	2 (8.0%)	2 (7.0%)
		United Kingdom	0 (0%)	0 (0%)	2 (7.0%)
		Tanzania	0 (0%)	1 (4.0%)	0 (0%)
	HCPs Years of experience	Mean	9.3 (±8.4)	5.2 (±4.9)	6.2 (±5.3)
	HCPs - How often treating neonate	Never or rarely	0 (0%)	4 (15.3%)	0 (0%)
		Sometimes	1 (20.0%)	2 (7.7%)	4 (13.3%)
		Frequently or very frequently	4 (80.0%)	20 (76.9%)	26 (86.7%)
Parents	Parent Hospital	MDH	3 (60.0%)	9 (75.0%)	4 (40.0%)
		CHUK	2 (40.0%	3 (25.0%)	6 (60.0%)
	Parent Social economic status	1–2 (Low)	1 (20.0%)	6 (50.0%)	3 (30.0%)
		2–4 (High)	4 (80.0%)	6 (50.0%)	7 (70.0%)
	Parental Residence	Urban	4 (80.0%)	12 (100%)	8 (80.0%)
		Rural	1 (20.0%)	0 (0%)	2 (20.0%)
	Parent Education	No education or primary	3 (60.0%)	6 (50.0%)	4 (44.4%)
		Secondary or Higher	2 (40.0%)	6 (50.0%)	5 (55.6%)

#### Round-1

In Round-1, during face-to-face interviews, the participants generated 20 education topics (Table 2). After coding, the topics were categorized into five themes; (i) Topics at admission; (ii) General neonatal care; (iii) Feeding; (iv) Cleanliness and Hygiene, and (v) Topics to be taught at the discharge period (Table 2). During Round-1 each topic was described by a mean of 4.0 participants (SD ± 2.0). Only three of the 20 (15.0%) topics were suggested by only one of the participants.

**Table 2:** Sections of the topics for the curriculum

Sections	Round-1	Round-2	Round-3	Round-3
	Total number of items	New items from participants	Total number of items presented in Round-3	Consensus criteria met
Topics at admission	6	8	14	8 (61.5%)
General care	4	8	12	10 (83.3%)
Feeding	6	7	13	11 (84.6%)
Cleanliness and hygiene	2	2	4	4 (100%)
Topics at discharge	2	12	14	14 (100%)
Total education topics	20	37	57	47 (83.9%)

#### Round-2

In Round-2 the participants generated 37 additional topics (Table 2) and these were again classified in the themes identified in Round-1. The 37 new topics were each described by a mean of 2.8 (±2.6) participants. Sixteen of the topics were suggested by only one of any of the stakeholders taking part in the Round (i.e. they were alone in suggesting that topic).

#### Round-3

In Round-3 the 20 and 37 topics from Rounds-1 and -2 respectively were combined, and the 57 items were ranked for importance by the participants. Forty-seven (84%) topics met the pre-defined consensus criteria to be included in the “parental neonatal curriculum” (Table 2).

### Parental neonatal curriculum

The topics were categorized into five domains representing the aspects of care of admitted newborns (Table 1 and Additional file 1). All the topics (except discharge topics) within our curriculum would be essential for any parent who is providing FICare of an admitted newborn in this setting. Topics on admission were generally about providing an “induction” for parents to the newborn care unit and explaining the reason for admission. Topics were then divided into the domains of “general care”, “feeding” and “hygiene” with “Kangaroo mother care”, “feeding quantity” and “hand washing” scoring the highest in each of these categories respectively. Finally, there were a series of topics that were considered to be important at the point of discharge, such as “follow up planning”. Nineteen of the topics in the curriculum were also specific to caregiving (e.g., feeding through a nasogastric tube).

### Correlation between groups

**Fig. 2:**
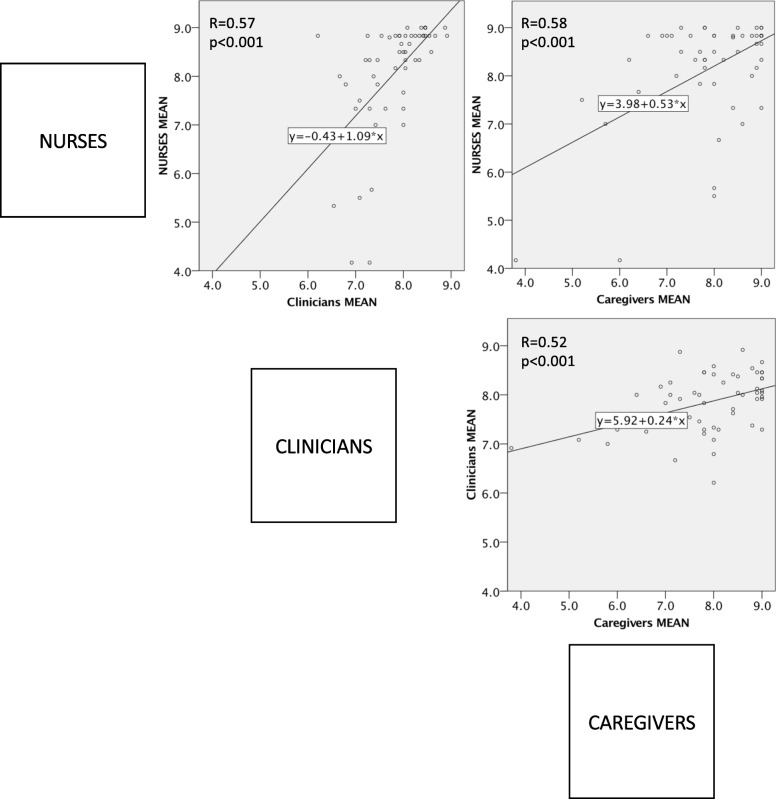
Correlation of Education topics between participants. *R = Pearson’s R correlation*

There was a moderate correlation in the importance of items between the three groups of stakeholders with Pearson’s R ranging between 0.52–0.57, *p* < 0.001 (Fig. 2). In Table 3 items have been presented with color coding to aid recognition of items with discordance in the opinion of stakeholders.

**Table 3:**
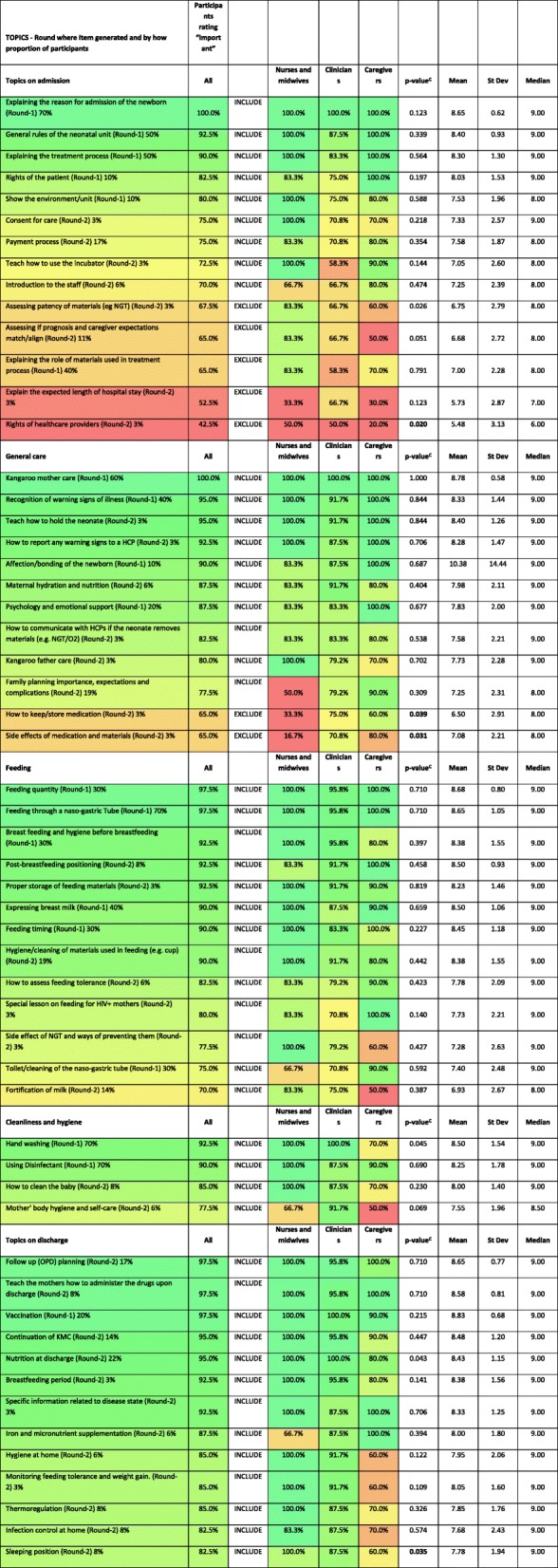
All education topics presented in Round-3

^C^Chi-squared *p*-value

## Discussion

This research project has identified the priority topics to be included in a “parental neonatal curriculum” for parents of admitted neonates in a resource-limited setting. The curriculum includes topics relating to admission, general care, feeding, cleanliness and hygiene, and discharge. This curriculum has been developed using robust Delphi-consensus techniques.

### Family integrated Care (FICare)

The admission of sick or preterm neonates is traumatic and stressful for parents; long-term it can cause impaired bonding and symptoms of post-traumatic stress disorder, affecting neonatal outcomes such as feeding [30]. FICare is a model of care that aims to reduce these adverse effects by integrating parents as primary caregivers of their sick newborns [8]. However, their integration needs to be underpinned by peer support and education; the Family-Led Care Model (FLCM) in Malawi is an excellent example of this [31]. FLCM was developed to improve facility- and home-based care of preterm/LBW newborns. This model enhances the skills of providers and the quality of care within KMC units, empowering families to directly participate in the care of their preterm/LBW newborn while still in the facility and with access to trained providers [32]. In Pakistan, it has been shown that it is possible to involve mothers in the active care of their very low birthweight infants before discharge and that this may translate into earlier discharge from the hospital without an increase in short term complications and readmission [33]. This FICare approach provides a cost-effective strategy for newborn units in resource-limited settings providing parents have been adequately educated.

### Healthcare professional competency and knowledge

Many parents will arrive on a neonatal unit with poor education levels and this can be a barrier to providing care [34]. In our stakeholder groups approximately 50% of participants had only primary or no formal education. It is also important to consider the level of knowledge and competencies in the healthcare professionals who may be educating parents on how to care for their sick, admitted, newborn. Ensuring that staff are well trained and have ongoing training to maintain competencies is essential for their own ability to care for neonates and to educate and support parents [34–36].

### Levels of agreement on curriculum items

Three groups of major stakeholders have been included here to gain a balanced curriculum to reflect the needs and wishes of each group of stakeholders. There was a moderate correlation in the importance of items between the three groups of stakeholders with Pearson’s R ranging between 0.52–0.57, *p* < 0.001 (Fig. 2). However, there were some topics where opinions varied markedly (Table 3). For example, parents had a strong wish for education on family planning which was not shared by nurses and clinicians. Even though there was some variation in opinions between stakeholder groups, only four of these were significantly different, and these four could also be explained by a large number of topics being tested without Bonferroni correction.

### Limitations

There is a lack of previous studies published on this particular subject to draw comparison. In Round-1 the face-to-face interviews were not audio-recorded. The purpose of Round-1 was not to identify rich theory (such as found in qualitative research), but rather to identify a preliminary list of topics. Audio-recording may have made participants reluctant to give full responses, fearful that they are being recorded. However, it is possible some topics were not documented in the field notes. The generalization of the study findings is limited because data collection occurred at just two hospital sites and in one geographical location. Another possible source of bias was acquiescence bias, the tendency of the participant to agree with statements or influential panel members, which we avoided in the initial rounds by explicitly avoiding the questions of agree/disagree and in later rounds by conducting a fully anonymized survey with no direct interaction between participants. Stakeholders tended to report that all the topics were “important”, resulting in only a small number of topics being excluded from the final curriculum. This could be explained by “respondent fatigue” in having to review 56 items for importance.

### Strengths

The strengths of this study include a high response rate from parents and clinicians and the broad experience of the HCPs who participated in this study. We followed a robust consensus methodology and have reported this work fully.

### Application of the findings

Any newborn care unit wanting to use the neonatal curriculum described here would be encouraged to tailor it their own patient population and their care needs. Our curriculum of topics was extensive, with 49 itemswhich may be beyond the capacity of a particular unit. The topics found in each domain of our curriculum have been ordered by “importance” given by the stakeholders so units may want to choose items which they feel are of “High”, “Medium” and “Low” priority based on their populations needs and on the capacity of their HCPs. Each subgroup of patients may also have distinct educational needs: the multiparous mother of an extremely preterm newborn may have very different educational needs to a primiparous mother of a near-term infant. There is no “one size fits all” educational package. Once parents have been given education on a curriculum item (e.g. nasogastric feeding) a competent HCP should assess the parental knowledge and skills before the specific task is undertaken by a caregiver.

### Future research in this area

Questions for further research include: what are the best methods to implement a curriculum of education for these parents, e.g. videos, group workshops, written literature, expert mothers, etc. We also did not investigate which parents should receive this curriculum to gain the maximum benefit. Education should enhance care, being implemented as part of a package to upscale care. Therefore, future research could potentially investigate the formal implementation of FICare in this setting, with parental education being a part of this package of care, and whether this is effective and cost-effective. Once implemented quantitative investigation of changes in care would be required as evidence in this setting is lacking. This may involve regular data collection through a Neonatal Registry [22] to allow for measures of change and loops of Quality Improvement [36]. Qualitative research would also be important to identify how individual populations want to receive education and which aspects they find most beneficial for caring for their admitted newborn.

## Conclusion

We have described a “parental neonatal curriculum,” formed using robust consensus methods. Greater improvement in neonatal care practices is essential if neonatal mortality reduction is to be achieved in resource-limited settings where the burden of disease is found. One step in achieving this could include the use of proven low-cost interventions such as FICare, based on a foundation of effective parental education.

## Additional files

Additional file 1: Parental neonatal curriculum. (DOCX 20 kb)

Additional file 2: Questions posed to participants. (DOCX 18 kb)

## Data Availability

The datasets generated and/or analyzed during the current study are not publicly available due to requiring consent from the approving ethical committee to share but are available from the corresponding author on reasonable request and approval from the relevant ethics board.

## Abbreviations

CHUK: University Teaching Hospital of Kigali
FICare: Family Integrated Care
HCPs: Health Care Professionals
HRH: Human Resources for Health
KMC: Kangaroo Mother Care
LBW: Low Birthweight
MoH: Ministry of Health
NMR: Neonatal Mortality Rate
