# Supplementary material for: What topics should we teach the parents of admitted neonates in the newborn care unit in the resource-limited setting - a Delphi study
Source: Matern Health Neonatol Perinatol. 2019 Jul 11;5:11. doi: 10.1186/s40748-019-0106-8 (PMC6621949; doi:10.1186/s40748-019-0106-8)
Supplement: Supplementary file 1 — Parental neonatal curriculum. (DOCX 20 kb) [file 40748_2019_106_MOESM1_ESM.docx]

**Supplementary file – Questions posed to participants**

|  |  | **Topics (curriculum)** |
| --- | --- | --- |
| **Round–1** | Parents | We are trying to identify the topics and subjects that parents need or want education on to better care for their unwell newborn.  What do you think are the key subjects you would like or need education on in order to better care for your baby at the hospital? |
|  | Expert stakeholders | We are trying to identify the topics and subjects that parents need or want education on to better care for their baby.  What do you think are the key topics that parents and caregivers need education in order to better care for their sick admitted neonates? Tell me as many as you can. |
| **Round-2** | Parents | “Imagine that you are caring for a sick neonate in the resource-limited setting. Parents and caregivers need education on how to better care for their sick newborn.  Please read through this list of topics that doctors, nurses and parents have previously told us that caregivers need.  Please list any additional topics or subjects that you would like to receive education on in order to better care for your sick newborn baby? |
|  | Expert stakeholders | “Imagine that you are caring for a sick neonate in the resource-limited setting. Parents and caregivers need education on how to better care for their sick newborn.  Please read through this list of topics that doctors, nurses and parents have previously told us that caregivers need to better care for their newborn baby.  Please list any additional topics or subjects that you would like to educate the parents on in order that they may better take care for their sick admitted neonate?” |
| **Round-3** | Parents | Imagine that you are caring for a sick neonate in the resource-limited setting. Caregivers undertake many tasks and duties for their sick newborn and need education to adequately undertake these tasks.  For each item, we will provide with you with the task, along with which round of the Delphi process it was reported and how frequently participants reported it.  QUESTION - Healthcare professionals are limited in the time they can spend educating you as a caregiver, how important is it that to you to receive education on the following "Topics at admission", "General care topics", "Feeding", "Cleanliness and hygiene", "Topics at discharge"? |
|  | Expert stakeholders | Imagine that you are caring for a sick neonate in the resource-limited setting. Caregivers undertake many tasks and duties for their sick newborn and need education to adequately undertake these tasks.  For each item, we will provide with you with the task, along with which round of the Delphi process it was reported and how frequently participants reported it.  QUESTION - Healthcare professionals are limited in the time they can spend educating caregivers. In your experience, how important is it that caregivers receive education on the following "Topics at admission", "General care topics", "Feeding", "Cleanliness and hygiene", "Topics at discharge"? |
