# Supplementary material for: What topics should we teach the parents of admitted neonates in the newborn care unit in the resource-limited setting - a Delphi study
Source: Matern Health Neonatol Perinatol. 2019 Jul 11;5:11. doi: 10.1186/s40748-019-0106-8 (PMC6621949; doi:10.1186/s40748-019-0106-8)
Supplement: Supplementary file 2 — Questions posed to participants. (DOCX 18 kb) [file 40748_2019_106_MOESM2_ESM.docx]

**Supplementary file: Parental neonatal curriculum**

| **TOPICS ON ADMISSION** |
| --- |
| 1. Explaining the reason for admission of the newborn |
| 1. General rules of the neonatal unit |
| 1. Explaining the treatment process |
| 1. Rights of the patient |
| 1. Show the environment/unit |
| 1. Consent for care |
| 1. Payment process |
| 1. How to use the incubator |
| 1. Introduction to the staff |
| **GENERAL CARE** |
| 1. Kangaroo mother care |
| 1. Recognition of warning signs of illness |
| 1. How to hold the neonate |
| 1. How to report any warning signs to a HCP |
| 1. Affection/bonding of the newborn |
| 1. Maternal hydration and nutrition |
| 1. Psychology and emotional support |
| 1. How to communicate with HCPs if the neonate removes materials (e.g. NGT/O2) |
| 1. Kangaroo father care |
| 1. Family planning importance, expectations and complications |
| **TOPICS ON FEEDING** |
| 1. Feeding quantity |
| 1. Feeding through a naso-gastric Tube |
| 1. Breast feeding and hygiene before breastfeeding |
| 1. Post-breastfeeding positioning |
| 1. Proper storage of feeding materials |
| 1. Expressing breast milk |
| 1. Feeding timing |
| 1. Hygiene/cleaning of materials used in feeding (e.g. cup) |
| 1. How to assess feeding tolerance |
| 1. Special lesson on feeding for HIV+ mothers |
| 1. Side effect of NGT and ways of preventing them |
| 1. Toilet/cleaning of the naso-gastric tube |
| 1. Fortification of milk |
| **CLEANLINESS AND HYGIENE** |
| 1. Hand washing |
| 1. Using Disinfectant |
| 1. How to clean the baby |
| 1. Mother' body hygiene and self-care |
| **TOPICS ON DISCHARGE** |
| 1. Follow up (OPD) planning |
| 1. Teach the mothers how to administer the drugs upon discharge |
| 1. Vaccination |
| 1. Continuation of KMC |
| 1. Nutrition at discharge |
| 1. Breastfeeding period |
| 1. Specific information related to disease state |
| 1. Iron and micronutrient supplementation |
| 1. Hygiene at home |
| 1. Monitoring feeding tolerance and weight gain |
| 1. Thermoregulation |
| 1. Infection control at home |
| 1. Sleeping position |
